# Integrated lipidomic and transcriptomic analyses identify altered nerve triglycerides in mouse models of prediabetes and type 2 diabetes

**DOI:** 10.1242/dmm.042101

**Published:** 2020-01-24

**Authors:** Phillipe D. O'Brien, Kai Guo, Stephanie A. Eid, Amy E. Rumora, Lucy M. Hinder, John M. Hayes, Faye E. Mendelson, Junguk Hur, Eva L. Feldman

**Affiliations:** 1 Department of Neurology, University of Michigan, Ann Arbor, MI 48109-2200, USA; 2 Department of Biomedical Sciences, University of North Dakota, Grand Forks, ND 58202-9037, USA

**Keywords:** Type 2 diabetes, Peripheral neuropathy, Triglyceride, Lipidomic, Saturated fatty acid, Diacylglycerol acyltransferase 2

## Abstract

Peripheral neuropathy (PN) is a complication of prediabetes and type 2 diabetes (T2D). Increasing evidence suggests that factors besides hyperglycaemia contribute to PN development, including dyslipidaemia. The objective of this study was to determine differential lipid classes and altered gene expression profiles in prediabetes and T2D mouse models in order to identify the dysregulated pathways in PN. Here, we used high-fat diet (HFD)-induced prediabetes and HFD/streptozotocin (STZ)-induced T2D mouse models that develop PN. These models were compared to HFD and HFD-STZ mice that were subjected to dietary reversal. Both untargeted and targeted lipidomic profiling, and gene expression profiling were performed on sciatic nerves. Lipidomic and transcriptomic profiles were then integrated using complex correlation analyses, and biological meaning was inferred from known lipid-gene interactions in the literature. We found an increase in triglycerides (TGs) containing saturated fatty acids. In parallel, transcriptomic analysis confirmed the dysregulation of lipid pathways. Integration of lipidomic and transcriptomic analyses identified an increase in diacylglycerol acyltransferase 2 (DGAT2), the enzyme required for the last and committed step in TG synthesis. Increased DGAT2 expression was present not only in the murine models but also in sural nerve biopsies from hyperlipidaemic diabetic patients with PN. Collectively, these findings support the hypothesis that abnormal nerve-lipid signalling is an important factor in peripheral nerve dysfunction in both prediabetes and T2D.

This article has an associated First Person interview with the joint first authors of the paper.

## Abstract (precis)

**Summary:** Mouse models of prediabetes and type 2 diabetes that develop peripheral neuropathy display increased levels of nerve triglycerides, which return to normal upon dietary reversal, suggesting that altered lipids are involved in disease.

## INTRODUCTION

Peripheral neuropathy (PN) is a common prediabetes and type 2 diabetes (T2D) complication characterized by length-dependent nerve loss in a distal-to-proximal fashion in affected limbs. PN is accompanied by numbness or positive sensory sensations, such as tingling or prickling, but ultimately results in complete loss of feeling (Feldman et al., 2019). Accumulating evidence suggests that hyperglycaemia alone is not responsible for PN, but that dyslipidaemia also plays a critical role in pathogenesis (Smith and Singleton, 2013). Abnormal circulating triglyceride (TG) levels correlate with PN progression and nerve dysfunction in prediabetes and T2D (Smith and Singleton, 2013; Wiggin et al., 2009). Moreover, obesity and systemic dyslipidaemia are independent risk factors for PN in obese and T2D patients (Callaghan et al., 2018; Callaghan et al., 2016a,b; Wiggin et al., 2009). What is less well understood, however, is whether specific lipid classes and levels in the nerve are impacted during PN pathogenesis in prediabetes and T2D.

Genetic T2D mouse models consistently develop PN and have proved invaluable for identifying potential mechanisms that underlie PN (Hur et al., 2015; O'Brien et al., 2015). Specifically, our transcriptomic analyses revealed that dysregulated lipid metabolism in peripheral nerves is a shared pathway across *db/db* and *ob/ob* T2D models (McGregor et al., 2018). Furthermore, using untargeted lipidomics, we identified patterns of altered lipid metabolism in sciatic nerve (SCN) from *db/db* mice (Sas et al., 2018), suggesting lipid dysfunction in PN. However, these findings are confounded by the genetic manipulation of leptin signalling in *db/db* mice.

To overcome the limitations of genetic models, we turned to diet-induced models. The C57BL/6J mouse fed a lard-based high-fat diet (HFD) rich in saturated fatty acids (SFAs) serves as a model of prediabetes-induced PN (Hinder et al., 2017; Vincent et al., 2009). HFD-fed C57BL/6J mice treated with low-dose streptozotocin (STZ) serve as a model of T2D-induced PN (O'Brien et al., 2018b). Both models consistently and reproducibly develop obesity and dyslipidaemia with increased thermal latencies, reduced nerve conduction velocities (NCVs) and loss of intraepidermal nerve fibre density (IENFD), characteristic of human PN (Hinder et al., 2017; Vincent et al., 2009). Interestingly, placing HFD and HFD-STZ mice back on a standard diet (SD) restores nerve function, independent of glycaemic status (Hinder et al., 2017; O'Brien et al., 2018a). These findings support the idea that dietary factors, including lipids, contribute to PN, and that improving metabolic health can restore nerve function. Yet, the specific lipid species in the peripheral nerves of HFD and HFD-STZ mice that are differentially regulated and may play a role in nerve damage remain unknown.

**Fig. 1.**
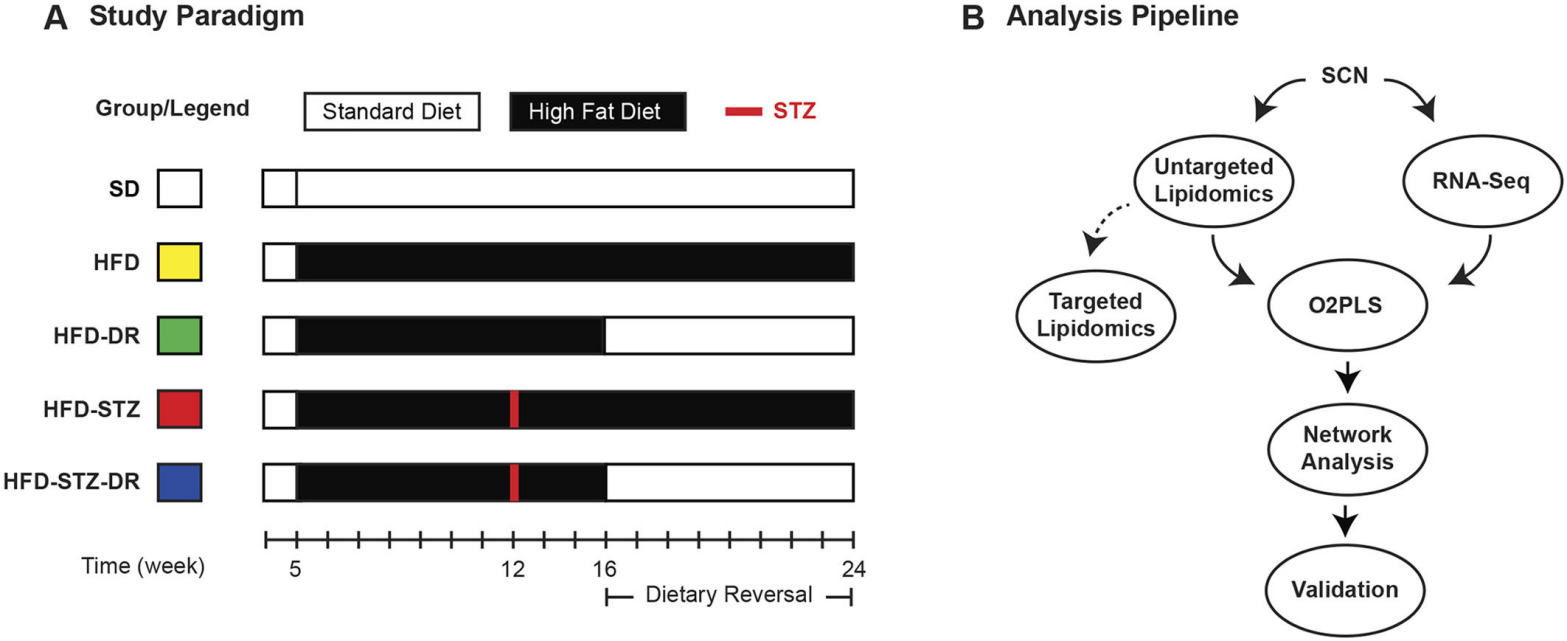
**Study overview and analysis pipeline.** (A) Experimental design. Animals began HFD at 5 weeks of age; two cohorts were administered STZ at 12 weeks of age to induce a T2D phenotype. At 16 weeks, a cohort of HFD and HFD-STZ mice were placed on the SD for 8 weeks. Animals were phenotyped and euthanized at baseline (16 weeks) and at study conclusion (24 weeks). The colours to the left relate to the colours used in subsequent figures. (B) Sciatic nerve (SCN) tissue collected at 16 and 24 weeks of age was processed for lipidomics or transcriptomics (*n*=9-10). To validate the untargeted lipidomics approach and to determine FA composition, TLC-GC was performed on a separate group of animals. O2PLS was used to integrate the lipidomic and transcriptomic datasets to identify key lipids and genes involved in PN development. A shared lipid-gene correlation network was also generated to pinpoint important lipids and genes. O2PLS, orthogonal partial least squares.

The aim of this study was to explore the impact on the class and levels of neural lipids and lipid metabolism in the SCN from a Western-style diet rich in SFAs in prediabetes (HFD) and T2D (HFD-STZ) mice along with a dietary reversal (DR) paradigm (HFD-DR and HFD-STZ-DR). Specifically, we phenotyped these mouse models for PN before and after DR, performed lipidome and transcriptome profiling of SCNs (Fig. 1A) and carried out comprehensive bioinformatic analyses (Fig. 1B). Nerve lipidomic and transcriptomic datasets were integrated using correlation and network analyses, and results cross-referenced with published lipid-gene interactions. Key lipids and genes associated with PN were further validated in sural nerve biopsies collected from patients with T2D and PN to support biological and clinical significance. Our findings reveal important dynamic changes in the nerve lipidome and transcriptome that highlight a potential involvement of TGs in PN pathogenesis and provide a better understanding of lipid homeostasis in the peripheral nervous system during prediabetes and T2D.

## RESULTS

### DR in HFD and HFD-STZ mice corrects PN

To gain insight into pathogenic changes along a continuum of metabolic disease, our study consisted of control mice on SD and two disease models, HFD and HFD-STZ, as well as the disease models placed on DR for 8 weeks (HFD-DR and HFD-STZ-DR, respectively) (Fig. 1A). Baseline metabolic and PN phenotyping was performed at 16 weeks of age. HFD mice consistently and reproducibly develop a prediabetic phenotype, including increased body weight (BW), impaired glycaemic control [elevated fasting blood glucose (FBG) and glycated haemoglobin (HbA_1c_), and impaired glucose tolerance] and dyslipidaemia (Fig. S1A-F), as we have previously reported in other experimental cohorts (Hinder et al., 2017; O'Brien et al., 2018a). HFD-STZ demonstrated a similar profile, although with more pronounced hyperglycaemia, recapitulating a more T2D phenotype (Fig. S1B). Both HFD and HFD-STZ mice displayed PN, with decreased sensory and motor NCVs, increased hindpaw withdrawal latency and decreased IENFD (Fig. S1G-J), as shown in earlier groups (Hinder et al., 2017; O'Brien et al., 2018a). Despite the pronounced hyperglycaemia in HFD-STZ mice, there was no difference in PN phenotype between disease models, a phenomenon we have recently reported (O'Brien et al., 2018b).

**Fig. 2.**
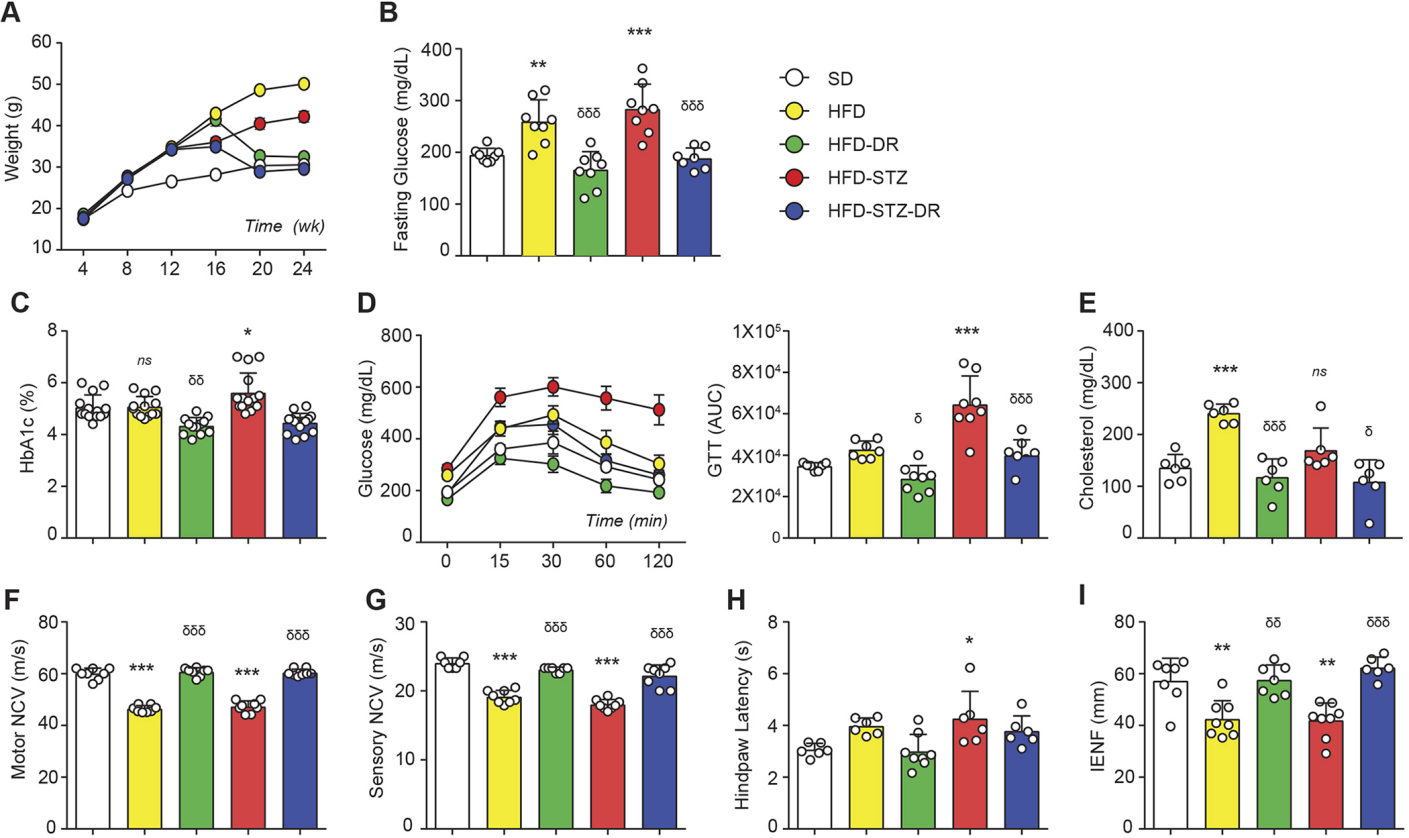
**Dietary reversal (DR) corrected metabolic and neuropathic phenotypes in mouse models of neuropathy.** (A) Longitudinal body weight (BW) taken every 4 weeks (*n*=15/16). (B) Fasting blood glucose (FBG) (*n*=7-8). (C) Percent HbA_1c_ (*n*=13-15). (D) Left: glucose tolerance test (GTT) on 4-h-fasted mice at 24 weeks of age following glucose injection (2 g/kg BW i.p.; *n*=8). Right: area-under-the-curve analysis for GTT (*n*=7-8). (E) Plasma cholesterol (*n*=6). (F) Motor nerve conduction velocity (NCV) (*n*=8). (G) Sensory NCV (*n*=8). (H) Hindpaw withdrawal latency (*n*=6-8). (I) Intraepidermal nerve fibre density (IENFD) (*n*=6-8). All measures above were performed at 24 weeks of age. *P<0.05, **P<0.01, ***P<0.001, one-way ANOVA followed by Tukey's multiple comparisons of diabetic (HFD/HFD-STZ) versus non-diabetic (SD) mice; ^δ^*P*<0.05, ^δδ^*P*<0.01, ^δδδ^*P*<0.001, one-way ANOVA followed by Tukey's multiple comparisons of diabetic mice placed on dietary reversal (HFD-DR/HFD-STZ-DR) versus diabetic (HFD/HFD-STZ). Data are presented as the mean±s.e.m.

The metabolic (Fig. 2A-E) and neuropathy (Fig. 2F-I) phenotypes of HFD and HFD-STZ mice persisted through 24 weeks of age. Eight weeks of DR (60% SFAs in HFD to 10% in SD) attenuated the metabolic abnormalities in both models, with improved BW, glycaemic control, and cholesterol levels (Fig. 2A-E). DR improved PN measures to a similar degree in HFD-DR and HFD-STZ-DR groups despite poorer glycaemic control in HFD-STZ mice (Fig. 2F-I).

### Untargeted lipidomics reveals distinct differences in TG between ‘disease’ and ‘reversal’ groups

**Fig. 3.**
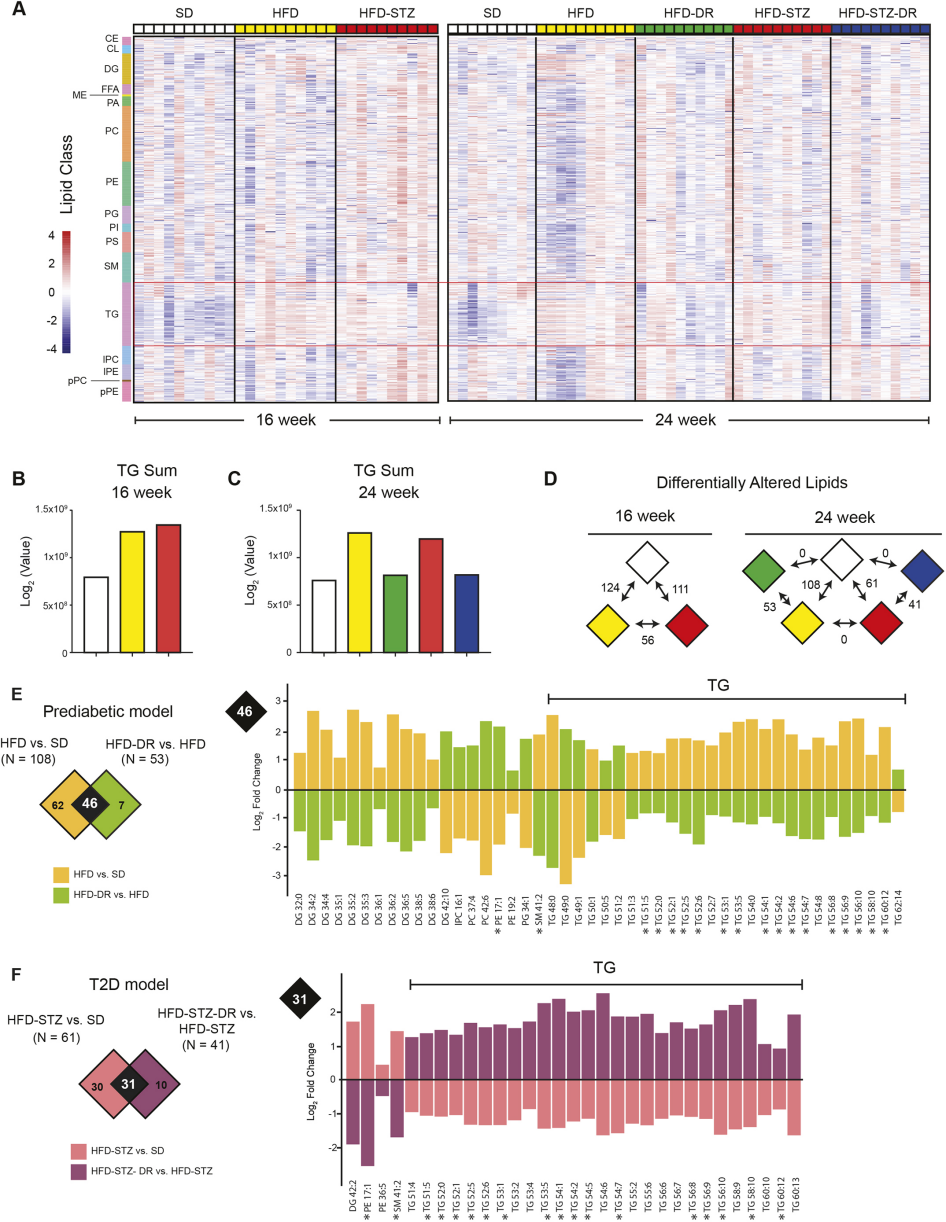
**Nerve TG levels correlated with neuropathy phenotype.** (A) Untargeted shotgun lipidomics was performed on SCN tissue isolated at 16 (left) and 24 (right) weeks of age. Differences in TG were observed when comparing groups at either time point (red outline) (*n*=9-10). The scale bar represents lipid levels that were *z*-score transformed at each lipid species. (B,C) The sum of the TG was determined at the (B) 16- or (C) 24-week time point. (D) The number of significant DALs was determined between groups at the 16- or 24-week time point (adjusted *P*-value=0.05). (E) To determine significant DALs in the prediabetic model, the HFD versus SD diabetic dataset (orange) was compared to the HFD-DR versus HFD reversal dataset (lime) (left panel). The overlapping DALs were plotted according to log_2_-fold change (right panel). (F) To determine significant DALs in the type 2 diabetic (T2D) model, the HFD-STZ versus SD diabetic dataset (salmon) was compared to the HFD-STZ-DR versus HFD-STZ reversal dataset (purple) (left panel). The overlapping DALs were plotted according to log_2_-fold change (right panel). Lipids that were common in both prediabetic and T2D models are denoted by an asterisk (*). CE, cholesteryl esters; CL, cardolipins; DAL, differentially altered lipid; DG, diglycerides; FFA, free fatty acids; ME, methyl esters; PA, phosphatidic acids; PC, phosphatidylcholines; PE, phosphatidylethanolamines; PG, phosphatidylglycerols; PI, phosphatidylinositols; PS, phosphatidylserines; SM, sphingomyelins; TG, triglycerides; LPC, lysophosphatidylcholines; LPE, lysophosphatidylethanolamines; pPC, plasmenyl-phosphatidylcholines; pPE, plasmenyl-phosphatidylethanolamines.

We next determined the nerve lipidome associated with PN pathogenesis in the setting of prediabetes and T2D with untargeted lipidomics, which identified 578 individual lipids in the SCN. Using basic lipid-class-based clustering and heat maps, a pattern emerged that demonstrated elevated TGs at 16 weeks of age in HFD and HFD-STZ nerves compared to mice on SD (Fig. 3A,B). Elevated TGs persisted through 24 weeks of age but were normalized with DR (Fig. 3C). This pattern was also observed to a lesser extent in cholesterol–fatty-acid ester (CE), diglyceride (DG), methyl ester (ME) and sphingomyelin (SM) lipid classes (Fig. S2). Our data suggest dysregulation of nerve lipids, particularly TGs, in PN pathogenesis.

We next used our standard bioinformatics pipeline to further interrogate lipidome changes associated with disease development. Unlike clustering and basic class analysis, this approach identifies individual lipids that are significantly altered between groups [differentially altered lipids (DALs)] to identify biologically relevant changes (Fig. 3D). To assess how DR reverses PN, we compared SD to HFD and SD to HFD-STZ (control versus disease), and HFD to HFD-DR and HFD-STZ to HFD-STZ-DR (disease versus reversal), to uncover shared and distinct lipid patterns (Fig. 3E,F). In our prediabetic model, we identified 46 common DALs (Fig. 3E), of which 12 were increased with HFD and 34 decreased by DR. In the T2D model, 31 DALs overlapped, of which four increased with HFD-STZ and 27 decreased in HFD-STZ-DR (Fig. 3F). Eighteen DALs were common between both models, 16 of which were TGs (Fig. 3E,F, * denotes shared lipids), suggesting that TGs are dysregulated during PN.

### SFAs are elevated in SCN of prediabetic mice

**Fig. 4.**
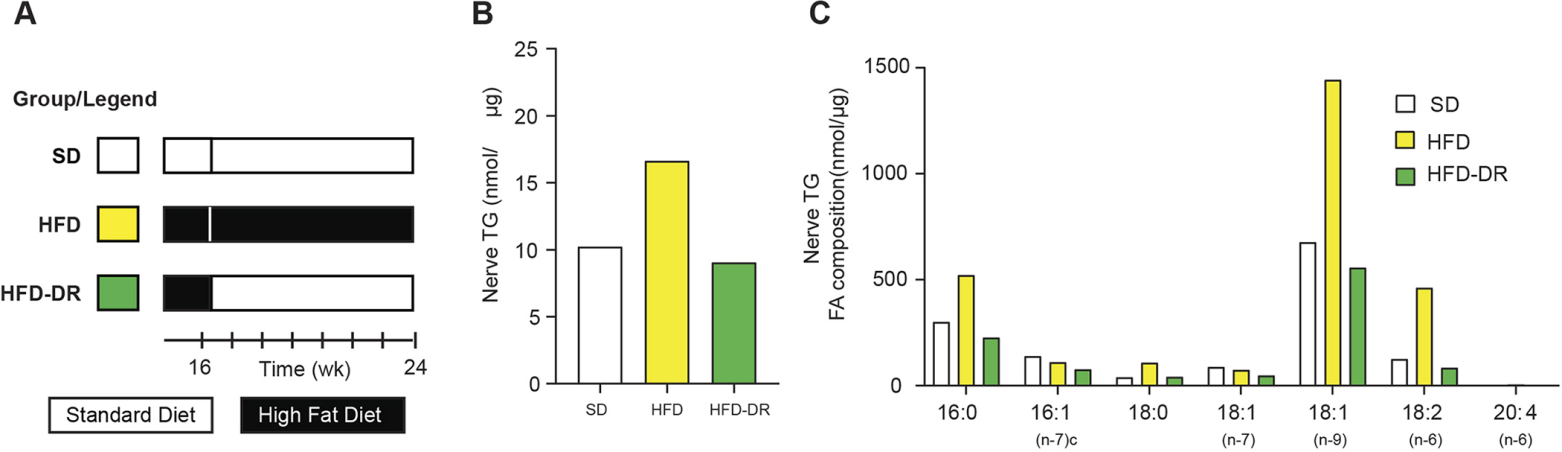
**SCN tissue derived from HFD mice has increased TG and FA levels.** (A) Additional mice were acquired to validate the findings from the untargeted lipidomic analysis. For each group, SCN tissue (*n*=10) was pooled before being subjected to TLC-GC. (B) Sum of TG. (C) Sum of the FA composition.

Thin layer chromatography (TLC)-gas chromatography (GC) (TLC-GC) was performed on SCNs from a separate cohort of SD, HFD and HFD-DR mice at 24 weeks of age to quantitate the untargeted lipidomics results (Fig. 4A). This assay confirmed our untargeted lipidomics class-based analysis: upregulation of TG content with HFD and normalization with DR (Fig. 4B). Analysis of specific fatty acids (FAs) comprising nerve TGs identified increased SFAs – palmitate (C16:0) and stearate (C18:0) – compared to SD and HFD-DR (Fig. 4C). A similar trend was observed for the unsaturated FAs oleate (C18:1) and linoleate (C18:2), but there was no change in palmitoleate (C16:1) levels. These results suggest that the FAs elevated in HFD chow (Table S1) are incorporated into the nerve TGs of HFD-fed mice.

### Transcriptome profiling identifies highly enriched lipid-related pathways

**Fig. 5.**
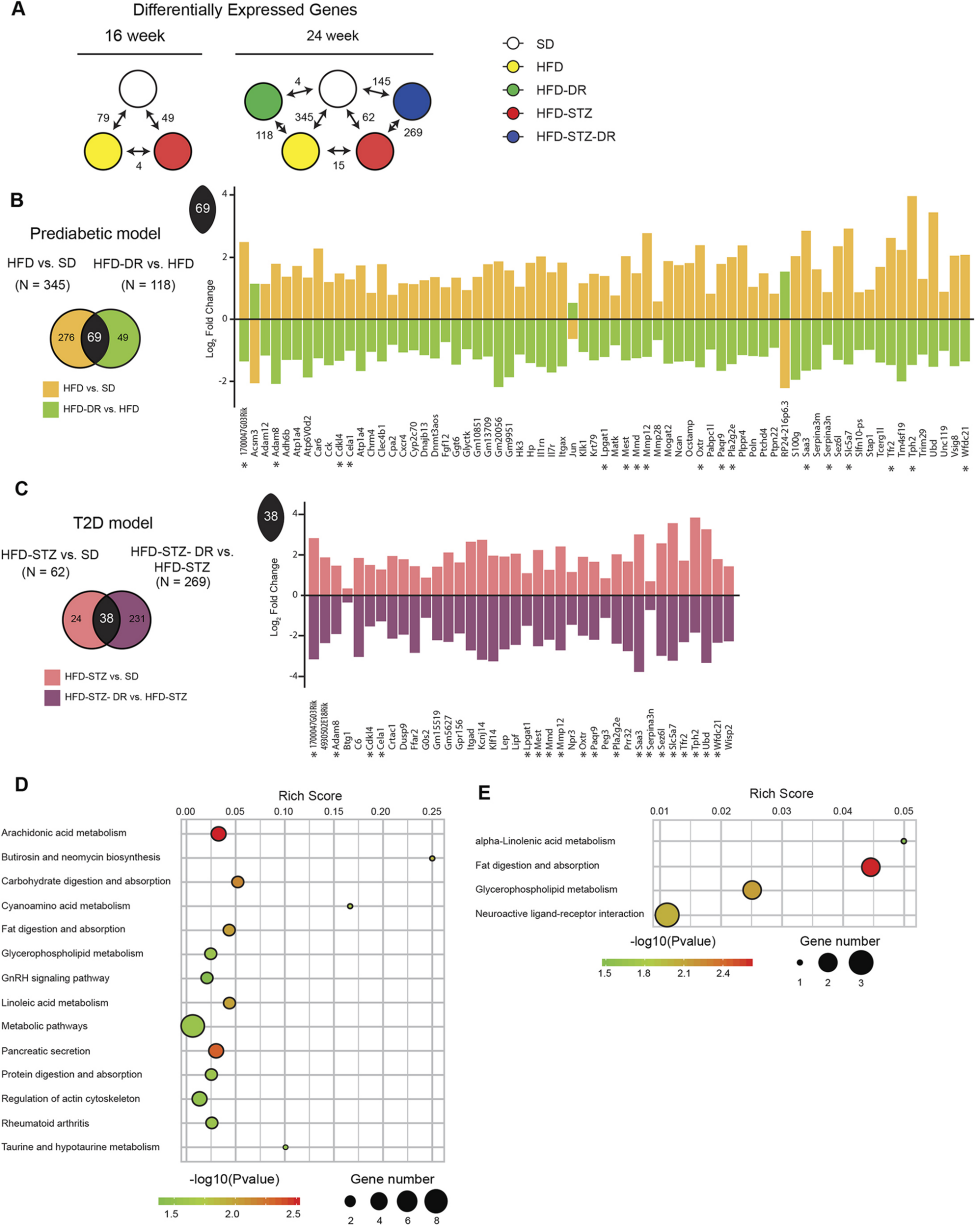
**Transcriptomics data analysis revealed lipid metabolism pathways to be significantly altered.** (A) The number of significant DEGs was determined between groups at the 16- or 24-week time point (adjusted *P*-value<0.05). (B) To determine significant DEGs in the prediabetic model, the HFD versus SD diabetic dataset (orange) was compared to the HFD-DR versus HFD reversal dataset (lime). (C) To determine significant DEGs in the T2D model, the HFD-STZ versus SD diabetic dataset (salmon) was compared to the HFD-STZ-DR versus HFD-STZ reversal dataset (purple). Gene transcripts that were common in both prediabetic and T2D models are denoted by an asterisk (*). (D,E) Functional enrichment analysis using KEGG pathways was performed on the 69 common DEGs found in the prediabetic model (D) or the 38 common DEGs found in the T2D model (E). Overlapping DEGs were plotted according to log_2_-fold change. DEG, differentially expressed gene; KEGG, Kyoto Encyclopedia of Genes and Genomes.

To complement our lipidomic analyses, transcriptomic changes were assessed by RNA-seq in SCN using a similar pipeline to that above (Fig. 5A). At 16 weeks of age, the prediabetes and T2D datasets contained 79 and 49 differentially expressed genes (DEGs), respectively, whereas, at 24 weeks of age, they contained 345 and 64 DEGs, respectively. Comparing disease to DR datasets at 24 weeks of age, 69 and 38 DEGs were identified in the HFD and HFD-STZ groups, respectively (Fig. 5B,C).

To identify key pathways associated with PN pathogenesis, functional enrichment analysis was performed on the common prediabetes and T2D DEGs using Kyoto Encyclopedia of Genes and Genomes (KEGG; https://www.genome.jp/kegg/) pathway analysis. Fourteen KEGG functions/terms were enriched among the 69 prediabetes DEGs, while four terms were enriched among the 38 T2D DEGs (Fig. 5D). Common KEGG terms were identified across both disease models: ‘fat digestion and absorption’ (two genes; *Lipf*, *Pla2g2e*) and ‘glycerophospholipid metabolism’ (two genes; *Pla2g2e*, *Lpgat1*). These analyses further support that lipid metabolism is dysregulated during PN pathogenesis.

### Integration of lipidome and transcriptome datasets reveals highly intercorrelated lipids and transcripts

**Fig. 6.**
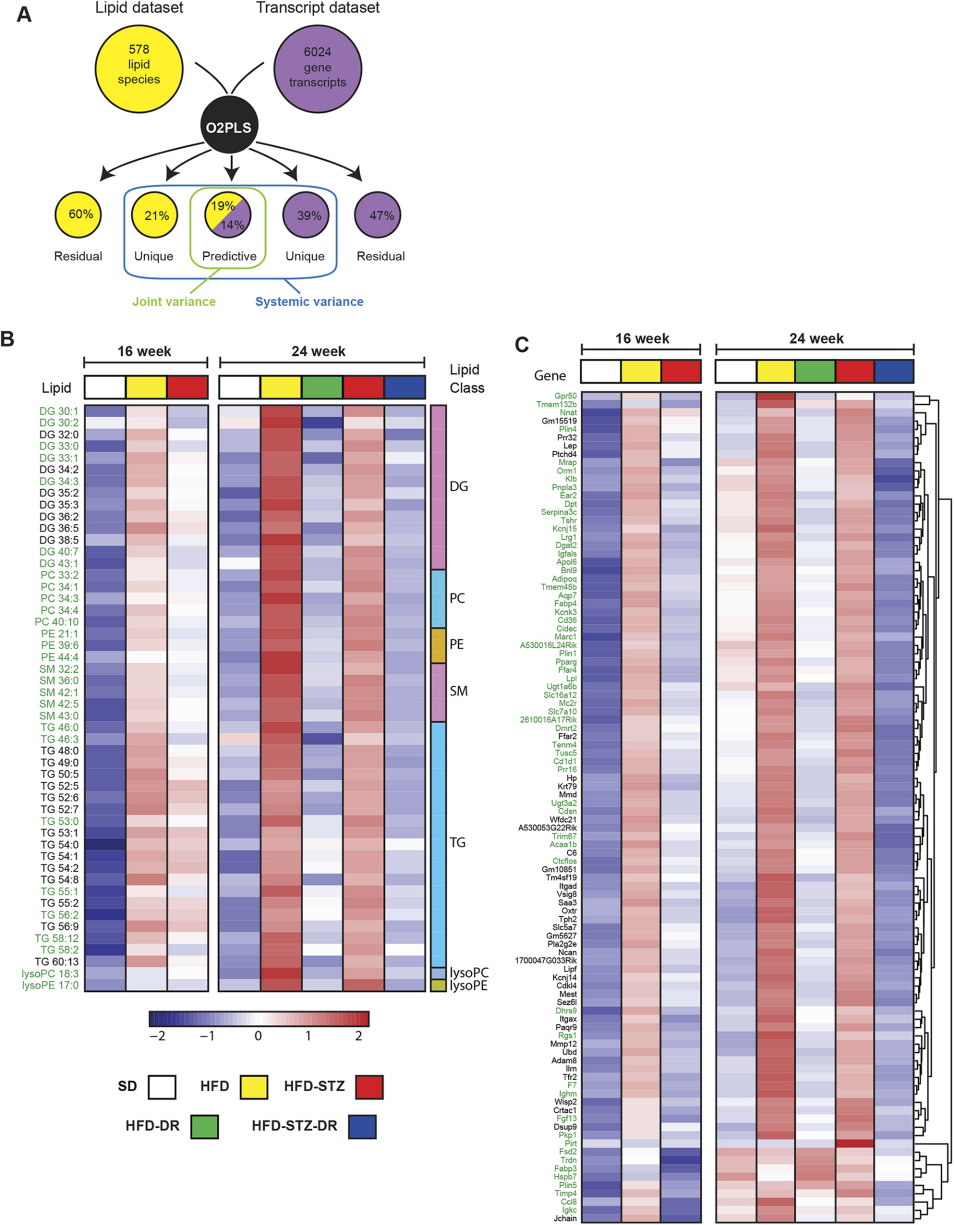
**Integrative analysis of transcriptome and lipidome.** (A) Overview of the O2PLS integrative analysis: each model structure shows the percentage of the total variance explained with a total of six components identified. Heat maps representing the average expression profiles of the top (B) 50 lipids and (C) 100 genes by O2PLS across each of the groups at 16 and 24 weeks of age. Lipids and genes identified only by O2PLS are written in green, while lipids and genes previously identified as common DALs/DEGs through our differential analysis are in black. DAL, differentially altered lipid; DEG, differentially expressed gene. DG, diglycerides; PC, phosphatidylcholines; PE, phosphatidylethanolamines; SM, sphingomyelins; TG, triglycerides; lysoPC, lysophosphatidylcholines; lysoPE, lysophosphatidylethanolamines.

As a first step towards integrating the lipidomic and transcriptomic datasets, 578 lipids and 6024 genes were selected using orthogonal partial least squares (O2PLS) to identify the joint covariation between the two datasets (Fig. 6A). Figure S3 illustrates predicted interactions between the lipid and transcript datasets in a scatter plot. The top 50 lipid (Table S2) and 100 gene (Table S3) candidates from this correlation analysis are highlighted in loading plots (Fig. S4). Novel lipids identified by O2PLS but not by DAL/DEG analysis were predominantly TGs and DGs, and novel genes include *Lpl* (lipoprotein hydrolysis), *Cd36* (FA uptake) and *Dgat2* (diacylglycerol O-acyltransferase 2; TG synthesis). A heat map of the top lipids and transcripts reveals distinctions between the SD, HFD, HFD-STZ, HFD-DR and HFD-STZ-DR groups (Fig. 6B,C), which become more pronounced following a longer duration of metabolic disease (Fig. S3).

### Lipid-transcript correlation network

**Fig. 7.**
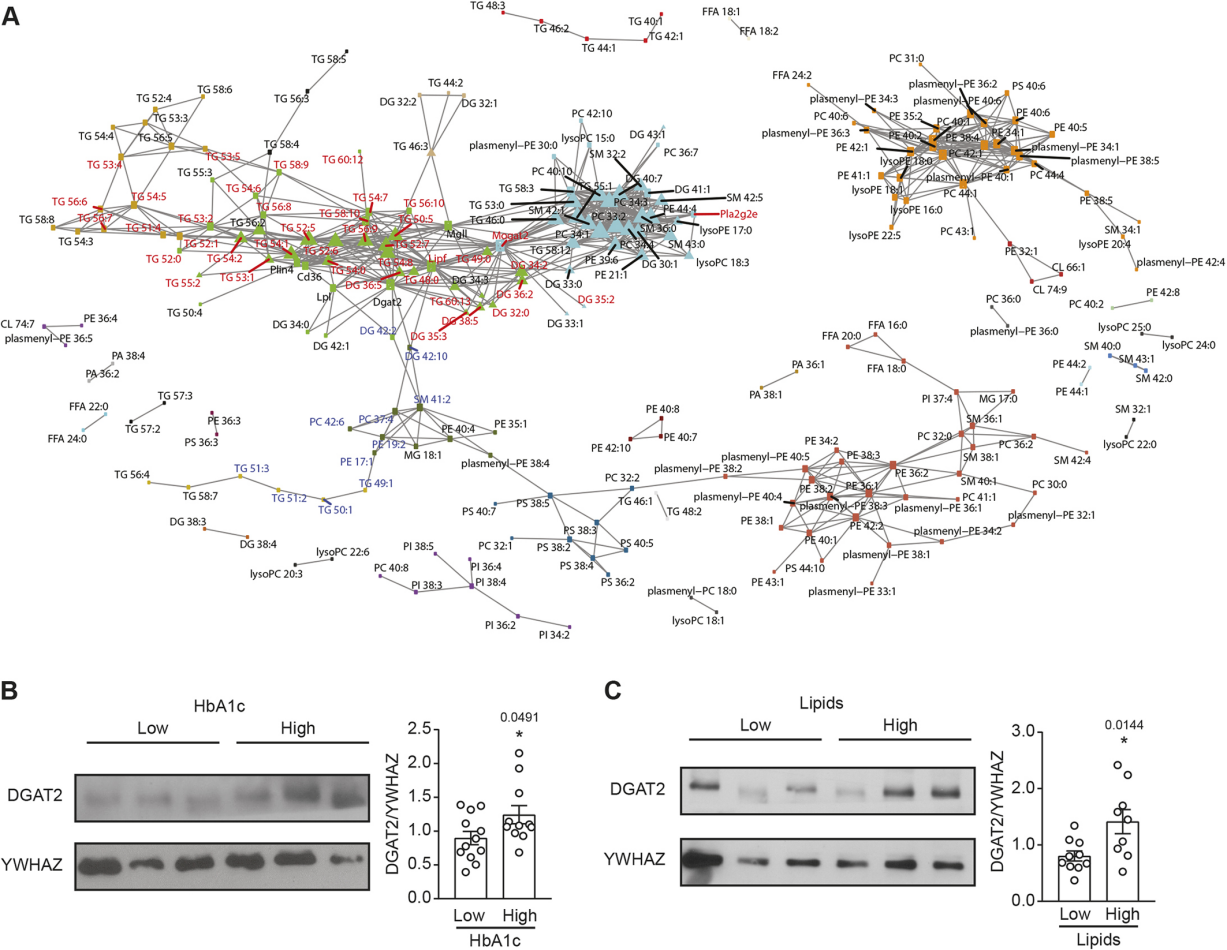
**DGAT2, identified in a lipid-gene transcript network, was elevated in sural nerve biopsy from patients with high lipid status.** (A) Lipid-gene transcript correlation network. A pairwise correlation network was constructed using 578 lipids and 92 genes, included in the O2PLS analysis, that had an annotated association in the HMDB. Only lipid-lipid and lipid-gene pairs with a Pearson correlation coefficient >0.6 or <−0.6 were included. Node=lipids and genes. Edge=correlated pair. Node colour=subnetworks identified by igraph. Node shape=type of entities (triangle=top lipid species from O2PLS; circle=lipids; square=genes with known lipid interactions). Node font colour=DR [black=not reversed; red=reversed (upregulated in HFD or HFD-STZ); blue=reversed (downregulated in HFD or HFD-STZ)]. (B) Left: representative immunoblot of microsome-derived lysates from sural nerve biopsies when patients were stratified according to HbA1c status. Right: densitometry analysis showing an increase in DGAT2 in the sural nerve of patients with high HbA1c. (C) Left: representative immunoblot of microsome-derived lysates from sural nerve biopsies when patients were stratified according to lipid status. Right: densitometry analysis showing an increase in DGAT2 in the sural nerve of patients with high lipids (elevated plasma cholesterol and TG). YWHAZ was used as the protein loading control. *n*=9/10. *P<0.05, two-tailed Student's *t*-test. Data are presented as the mean±s.e.m. DR, dietary reversal; HMDB, Human Metabolome Database.

To filter the identified pairs of highly correlated lipids and transcripts for biological significance, we employed a correlation network analysis. This network was constructed from 578 lipids and 92 genes with known lipid association according to the Human Metabolome Database (HMDB), using the previously identified highly correlated pairs. The final lipid-transcript correlation network included 231 lipids and eight genes, with 31 highly interconnected subnetworks (Fig. 7A), of which four were large and contained 24 or more nodes (approximately 10% of total nodes). All eight genes were connected to two subnetworks comprising mostly TGs and DGs (Fig. 7A, green) or various lipids (Fig. 7A, cyan). Of note, most of the lipids in the green subnetworks were significantly affected by prediabetes and/or T2D but reversed by DR, suggesting an association of TGs and DGs with PN pathogenesis. Six of the eight genes were also present in the top 100 O2PLS candidate transcripts (including *Lpl*, *Cd36* and *Dgat2*) (Table S3), further supporting their dysregulation in nerve lipid homeostasis.

To validate our findings, we determined the expression levels of the six genes that overlapped between O2PLS and network correlation analyses using quantitative real-time PCR (qPCR) on pooled archived RNA from SCNs from 24-week-old SD, HFD and HFD-DR mice. Expression was increased in HFD compared to SD mice, and decreased in HFD-DR for all genes (Fig. S5), suggesting that the FA uptake and TG synthesis machinery is upregulated in the SCN of prediabetic mice.

### Increased DGAT2 protein in neuropathic patients with hyperlipidaemia

Our lipidomic findings implicate that increased TGs correlate with HFD-induced PN. Integration with transcriptomic data pinpoints *Dgat2* as a crucial gene by both O2PLS and network correlation analysis, which we validated by qPCR. DGAT2 catalyses the final and only committed step in TG synthesis (Choi et al., 2007) but, despite its central role in TG metabolism, its relevance to human PN is unknown. Therefore, we assessed DGAT2 protein expression in microsomes isolated from sural nerve biopsies of diabetic patients with PN who were stratified based on severity of metabolic disease (higher HbA_1c_ or TG and cholesterol levels; Tables S4 and S5, respectively). DGAT2 protein expression was increased in sural nerves of diabetic patients with high HbA_1c_ or high TG and cholesterol levels compared to diabetic patients with low HbA_1c_ or low TG and cholesterol levels (Fig. 7B,C). Importantly, elevated TG and cholesterol were associated with a loss of myelinated fibres, and therefore had a more severe PN phenotype (Table S5). Overall, these data suggest that DGAT2 expression correlates with PN pathogenesis and could be a potential target.

## DISCUSSION

Although several studies have shown that dyslipidaemia is associated with PN development, the precise pathogenic mechanisms remain unclear. We recently identified changes in lipid classes within the SCNs of *db/db* mice (Sas et al., 2018), but these findings were confounded by deficient leptin signalling. For diet-induced models, we recently performed a strain comparison analysis across three genetic backgrounds commonly used in diabetes research, BKS, BTBR and C57BL/6J. We identified HFD-fed C57BL/6J mice as the most robust experimental model of prediabetes, obesity and PN (Hinder et al., 2017). Here, we employed our recently published mouse models of prediabetes and T2D with PN on the optimal C57BL/6J background (O'Brien et al., 2018b), combined with a DR paradigm that reverses metabolic disease and PN (Hinder et al., 2017). These models are not genetically manipulated, parallel the human disease, and are clinically relevant. Using untargeted and targeted lipidomic profiling, we report changes in lipid classes and species in SCNs from animals with prediabetes and T2D-induced PN. Integration of lipidomic and transcriptomic datasets from SCNs of both disease models revealed that TGs and a TG synthesis pathway – possibly from diet-derived FAs – were associated PN pathogenesis. DGAT2 was identified as a key upregulated enzyme in HFD and HFD-STZ mice, which was also the case in sural nerve biopsy from hyperlipidaemic diabetes patients with PN. Our data provide the first comprehensive insight into dysregulated lipid metabolism pathways in peripheral nerves during metabolic disease and suggest that TG levels correlate with PN pathogenesis.

We have reported that dietary obesity coupled with impaired glucose tolerance are the primary metabolic factors linked to PN in four distinct clinical cohorts (Andersen et al., 2018; Callaghan et al., 2018, [Bibr DMM042101C5][Bibr DMM042101C5],b). Separate clinical trials, including Look AHEAD (Look AHEAD Research Group, 2017) and our Impaired Glucose Tolerance Study (Smith et al., 2006), demonstrated that diet and exercise improved PN and metabolic fitness in prediabetes and T2D patients. We recently reproduced these findings in HFD mice following DR (Hinder et al., 2017), providing a clinically relevant experimental model to study how DR ameliorates PN pathogenesis in prediabetes (Cooper et al., 2018; Coppey et al., 2018). In this study, we extend our findings to include a T2D model, the HFD-STZ mouse. Although HFD-STZ mice had higher FBG and HbA_1c_, there was no significant difference in PN phenotype compared to HFD mice, suggesting that nerve injury begins in the prediabetic state, before progression to overt T2D. These findings are in line with our published experimental and clinical data (Callaghan et al., 2016b; O'Brien et al., 2018b). Moreover, by implementing DR earlier (16 vs 20 weeks of age) and extending its duration (4 vs 8 weeks of age), both HFD and HFD-STZ mice displayed improved behaviour, NCVs and IENFDs compared to our previous findings (O'Brien et al., 2018b). ‘Replicating’ the human condition in these non-genetic mouse models allowed us to examine neural lipid homeostasis in models more relevant to the human disease.

In this study, we report for the first time that TGs on a class level are elevated in SCN at 16 and 24 weeks of age in HFD and HFD-STZ mice, and this is reversed by DR, suggesting an association of raised TG nerve content with PN. These changes are consistent with clinical and experimental evidence in support of a correlation between plasma TGs and PN development, further supporting a potential involvement of TGs in PN pathogenesis (Lupachyk et al., 2012; Smith and Singleton, 2013; Wiggin et al., 2009). We specifically observed an elevation in TG acyl chains derived from the SFAs palmitate (C16:0) and stearate (C18:0). Since dietary palmitate and stearate content is elevated in the HFD relative to the SD, these results suggest that diet-derived SFAs are incorporated into nerve TGs. The idea that these SFA species negatively impact peripheral nerve function is supported by reports that obesity linked to an SFA-rich diet correlates with PN development in patients with prediabetes and T2D (Callaghan et al., 2018, 2016a,b). Moreover, primary mouse sensory neurons exposed to SFAs have impaired mitochondrial function and bioenergetic capacity, both signs of axonal injury (Rumora et al., 2018, 2019). We also detected increased levels of TG acyl chains derived from the monounsaturated FA oleate in SCNs of HFD mice. Experimental and clinical studies reported beneficial effects of unsaturated FAs on nerve function (Coppey et al., 2018; Davidson et al., 2018; Shevalye et al., 2015; Yorek et al., 2017). The differential impact of oleate may be related to the lower ratio of dietary monounsaturated FAs to SFAs in the lard-based HFD chow (Coppey et al., 2018; Listenberger et al., 2003). Our findings may also relate to the source of dietary oleate, which is lard-based (animal), and may not be as beneficial to nerve function as plant-derived oleate (de Oliveira Otto et al., 2012; Wanders et al., 2017). This emphasises the importance of understanding both the source of the FA and the specific FA species constituting nerve TGs.

We also identified a role for genes involved in dysregulated lipid metabolism (*Lipf*, *Pla2g2e*) and sterile inflammation (*Saa3*, *Il1rn*) in PN using both transcriptomic and O2PLS analyses. These findings are consistent with our previous reports in *ob/ob* and *db/db* mouse models (Hur et al., 2015; O'Brien et al., 2015; Pande et al., 2011). The regulation of these pathways in SCNs from four murine models with PN (HFD, HFD-STZ, *ob/ob* and *db/db)* strongly suggests that loss of normal lipid metabolism and sterile inflammation are biologically meaningful with respect to PN pathogenesis. Parallel changes in these pathways are present in sural nerves from patients with diabetes and PN (McGregor et al., 2018), further highlighting the potential pathogenic relevance of altered nerve lipid metabolism and inflammation in PN.

When O2PLS integration was combined with correlation network analysis we continued to observe genes in the HFD and DR groups whose encoded proteins play critical roles in lipid pathways, including lipoprotein catabolism, FA transport and complex-lipid/TG synthesis. *Lipf* was again identified as a gene of interest along with *Lpl*, *CD36* and *DGAT2*. Lipoprotein lipase and gastric lipase (encoded by *Lpl* and *Lipf*, respectively) hydrolyses circulating lipoprotein TGs and gastrointestinal dietary TGs, respectively. While the significance of gastric lipase to PN is largely unknown, lipoprotein lipase is implicated in myelination (Rachana et al., 2018), and its upregulation in our models suggests dysregulation of myelin lipid homeostasis. CD36, a long-chain-FA transporter, mediates palmitate-induced apoptosis in the diabetic kidney (Susztak et al., 2005) and oxidized lipid toxicity in the diabetic retina (Fu et al., 2014). In agreement with our current data, we previously reported increased CD36 expression in nerves of *db/db* mice with PN (Pande et al., 2011) and in patients with progressive diabetic PN (Hur et al., 2011). Collectively, these results support future studies to identify potentially cohesive mechanisms underlying lipoprotein lipase- and CD36-mediated nerve injury in metabolic disease (Vincent et al., 2009), and their potential as therapeutic targets for PN treatment.

An important candidate identified by our integrative analyses was *Dgat2*, which is of particular interest because of its critical role in regulating lipid content by promoting TG synthesis (Choi et al., 2007). DGAT2 is a microsomal enzyme found mainly in the endoplasmic reticulum that catalyses the final and only committed step in TG synthesis by converting fatty acyl-CoA and diacylglycerol to TG (Choi et al., 2007). *Dgat2* expression is increased and contributes to damage in metabolically active tissues such as the heart, liver and adipose tissue in HFD mice (Ji et al., 2017; Roe et al., 2018; Suzuki et al., 2005). However, little is known about DGAT2 expression and function in PN. In the current study, we observed increased *Dgat2* in the SCN of HFD mice that was restored by DR. To determine whether DGAT2 dysregulation is conserved in human PN, we examined DGAT2 protein levels in sural nerve biopsies collected from patients with T2D and PN (Wiggin et al., 2009). Increased DGAT2 protein levels were associated with an abnormal lipid profile and worse PN phenotype. We also observed increased DGAT2 in T2D patients with poor glycaemic control. These results demonstrated that peripheral nerve TG homeostasis is dysregulated during PN pathogenesis in both mice and humans.

In this study, we identified increases in DGAT2 and TG levels in the SCN of HFD-fed mice with PN that are reversed by DR. Although these analyses capture lipidomic and transcriptomic changes that occur in 16- and 24-week-old mice with established PN, these analyses do not capture early changes that drive PN onset prior to 16 weeks of age. Future studies will examine the nerve lipidome and transcriptome in the early disease state to identify causal mediators of PN onset. We have recently identified sex differences in PN using *ob/ob* mice (O'Brien et al., 2016), which suggests that similar changes may occur in HFD C57 BL/6J mice. Although not part of the current study, we are currently investigating the effect of sex dimorphism on the development of HFD-induced PN. In addition, future work will also evaluate cell-specific changes within the SCN since it is comprised of Schwann cells and endothelial cells, along with neuronal axons. These future studies will enhance our understanding of the contributions of each specific cell type or nerve component to the development of PN in prediabetic and T2D murine models

In summary, we demonstrate reversal of PN using a simple dietary intervention in mice with prediabetes or T2D. We further demonstrate that hyperglycaemia is not a driver of PN along this continuum of metabolic disease. To understand mechanisms beyond hyperglycaemia, we integrated nerve lipidomic and transcriptomic profiles using correlation and network analyses. Our data collectively highlighted that nerve lipid metabolism was dysregulated and centred on DGAT2 and TG synthesis as being correlated with in PN pathogenesis in mice and humans. We suggest that HFD-derived SFAs are possibly incorporated into peripheral nerve TGs and may be involved in peripheral nerve injury. Future studies are required to determine exactly how these specific TG species impair peripheral nerve function and contribute to PN pathogenesis. Nonetheless, these data highlight the therapeutic efficacy of dietary intervention, aimed at reducing SFA-containing TGs, as a disease-modifying therapy for PN.

## MATERIALS AND METHODS

### Mice

Four-week-old male C57BL/6J mice (*n*=16/group; cat# 000664, The Jackson Laboratory, Bar Harbor, ME, USA) were randomised to SD, HFD, HFD-STZ, HFD-DR and HFD-STZ-DR cages with corn-cob bedding and water and food *ad libitum*. Cages were housed in a pathogen-free suite (20±2°C; 12:12 h light:dark cycle) and monitored daily by veterinarian staff. At 5 weeks of age, mice were switched to SD or HFD (D12450B and D12492, respectively; Research Diets, New Brunswick, NJ, USA) (Fig. 1). STZ was administered as described previously (O'Brien et al., 2018b). Briefly, mice received a first STZ dose at 12 weeks of age (75 mg/kg body weight, i.p.; cat# 329420; BD Biosciences, San Jose, CA, USA) followed 72 h later by a second dose (50 mg/kg body weight, i.p.). All procedures were approved by the University of Michigan Committee on Use and Care of Animals.

### Metabolic phenotyping

FBG levels, glucose tolerance test (GTT) and oxidized LDL (oxLDL) were measured as described previously (O'Brien et al., 2018b). Plasma insulin, cholesterol and TG lipoprotein profiles were assessed at the Mouse Metabolic Phenotyping Center (MMPC; www.mmpc.org) in a blinded manner.

### Neuropathy phenotyping

Thermal latency, sural sensory and sciatic-tibial motor NCVs, and IENFD were performed for neuropathy phenotyping as previously described (O'Brien et al., 2018b) in accordance with guidelines by the Diabetic Complications Consortium (www.diacomp.org).

### Untargeted lipidomics

Liquid chromatography tandem mass spectrometry (LC-MS/MS) untargeted lipidomics of SCN, including sample preparation and quality control, was performed by the Michigan Regional Comprehensive Metabolomics Resource Core (MRC2; www.mrc2.umich.edu) as previously described (Sas et al., 2018) in a blinded manner. Lipids were extracted from homogenized SCNs using a modified Bligh-Dyer method (Bligh and Dyer, 1959).

### Lipid identification and profiling

Lipids were identified using LipidBlast (http://fiehnlab.ucdavis.edu/projects/LipidBlast). Quantification was performed by MultiQuant software (SCIEX, Concord, Canada). Missing values were imputed using a K-nearest neighbour (KNN) algorithm and data were normalised using internal standards. Lipids identified in both positive and negative ion modes were merged into a mean value. Differential lipids were identified by Student's *t*-test between groups, with the Benjamini-Hochberg (BH)-corrected *P*<0.05 as the significance cut-off. The lipid species values from this output are provided in Table S6.

### Targeted lipidomics

Targeted lipidomics was performed by MRC2 in a blinded manner. Briefly, ten SCNs were combined, homogenised (Bligh and Dyer, 1959), and neutral lipids, including TG and diacylglycerols, were separated on a 20×20 cm TLC plate (Merck, Darmstadt, Germany) using a solvent system composed of hexane:diethyl ether:acetic acid (80:20:1, v/v) (Wood et al., 1969). GC was used to determine lipid levels by analysing the side-chain FA components from each lipid group using an HP-88 column (Agilent Technologies, Santa Clara, CA, USA) (Morrison and Smith, 1964). Each FA level was normalised to tissue weight and volume.

### RNA-seq

Total RNA was isolated from SCNs using an RNeasy Mini Kit (Qiagen, Germantown, MD, USA) and quality-assessed using a 2100 Bioanalyzer (Agilent Technologies). The sequencing library was prepared using SMART-Seq v4 Ultra Low Input RNA Kit with non-stranded and polyA+ selection (Clontech, Takara Bio USA, Mountain View, CA, USA). Approximately 120-million 125-bp paired-end reads per sample were obtained (HiSeq 2500, Illumina, San Diego, CA, USA). RNA-seq was performed by the University of Michigan DNA Sequencing Core (https://client-seqcore.brcf.med.umich.edu/) in a blinded manner. The raw data are deposited in the Gene Expression Omnibus database (www.ncbi.nlm.nih.gov/geo) under accession number GSE140201.

### Differential gene expression analysis

Low-quality (*Q*<30) reads and sequencing adapters were removed and clean reads were mapped to the mouse reference genome mm10 (GRCm38) using HISAT2 mapper (https://ccb.jhu.edu/software/hisat2/index.shtml) (Kim et al., 2015). FeatureCounts (http://subread.sourceforge.net/) summarised the reads mapped to mouse genes (Liao et al., 2014). Fragments per kilobase of transcript per million mapped reads (FPKM) values were calculated for all genes to represent their expression levels. DEGs were identified using DESeq2 (https://bioconductor.org/packages/release/bioc/html/DESeq2.html) (Love et al., 2014) with adjusted *P*<0.05 cut-off.

### Functional enrichment analysis

Significantly enriched pathways were determined using KEGG (https://www.genome.jp/kegg/) pathways, with a significance *P*<0.05 cut-off using RichR (https://github.com/hurlab/RichR).

### Two-way orthogonal partial least squares (O2PLS)

O2PLS analysis was used to integrate lipidomics and transcriptomics data to identity highly intercorrelated lipids and gene transcripts (Trygg and Wold, 2003). To reduce the computational complexity, analysis was limited to transcripts with potential differential signals. Lipidomics and transcriptomics data were scaled and transformed as previously described (Bylesjö et al., 2007). O2PLS analysis was performed using an R package (https://www.r-project.org/) (Bouhaddani et al., 2016), and the loading values for the joint covariance part were extracted to find highly interassociated genes and lipids.

### Lipid-transcript correlation network

A network of correlative lipids was constructed using the significant pairs with a Pearson correlation coefficient >0.6 or <−0.6. The network was extended by incorporating highly correlated transcripts with these lipids. The lipid-transcript associations were extracted from HMDB (http://www.hmdb.ca/), which included 316 genes associated with at least one of the lipids in the correlation network. This gene set was further filtered to include the O2PLS integrative analysis resulting in 92 genes. Subnetworks, highly interconnected based on the network topology, were identified with igraph (https://igraph.org/).

### qPCR validation

Biological validation was performed by qPCR (Hur et al., 2015), using sequence-specific primers (Table S7).

### Human sural nerve biopsies

Biopsies were obtained during a double-blind, placebo-controlled, 52-week clinical trial of acetyl-L-carnitine treatment for PN (Hur et al., 2011; Wiggin et al., 2009). Here, T2D patients were divided into two cohorts based on HbA_1c_ levels or cholesterol and TG levels (Tables S4 and S5).

### Microsome isolation

Microsomes were isolated from sural nerve biopsies with a Microsome isolation kit according to the manufacturer's instructions (Abcam, Cambridge, MA, USA).

### Immunoblotting

Immunoblotting was performed as described previously (Wiggin et al., 2009). A total of 20 µg of protein lysate was separated by SDS-PAGE and nitrocellulose membranes were incubated with a goat polyclonal antibody against DGAT2 (Abcam, cat# ab59493, RRID: AB_941282) and a rabbit polyclonal antibody against YWHAZ (Proteintech Group, cat# 14881-1-AP, RRID: AB_2218248).

### Statistical analyses

Statistical analyses were performed using Prism 7 (GraphPad Software, La Jolla, CA, USA) as previously described (O'Brien et al., 2018b).

This article is part of a special collection ‘A Guide to Using Neuromuscular Disease Models for Basic and Preclinical Studies’, which was launched in a dedicated issue guest edited by Annemieke Aartsma-Rus, Maaike van Putten and James Dowling. See related articles in this collection at http://dmm.biologists.org/collection/neuromuscular.

## Supplementary Material

Supplementary information
