# Supplementary material for: Integrated lipidomic and transcriptomic analyses identify altered nerve triglycerides in mouse models of prediabetes and type 2 diabetes
Source: Dis Model Mech. 2020 Jan 24;13(2):dmm042101. doi: 10.1242/dmm.042101 (PMC6994925; doi:10.1242/dmm.042101)
Supplement: Supplementary information [file dmm-13-042101-s1.pdf]

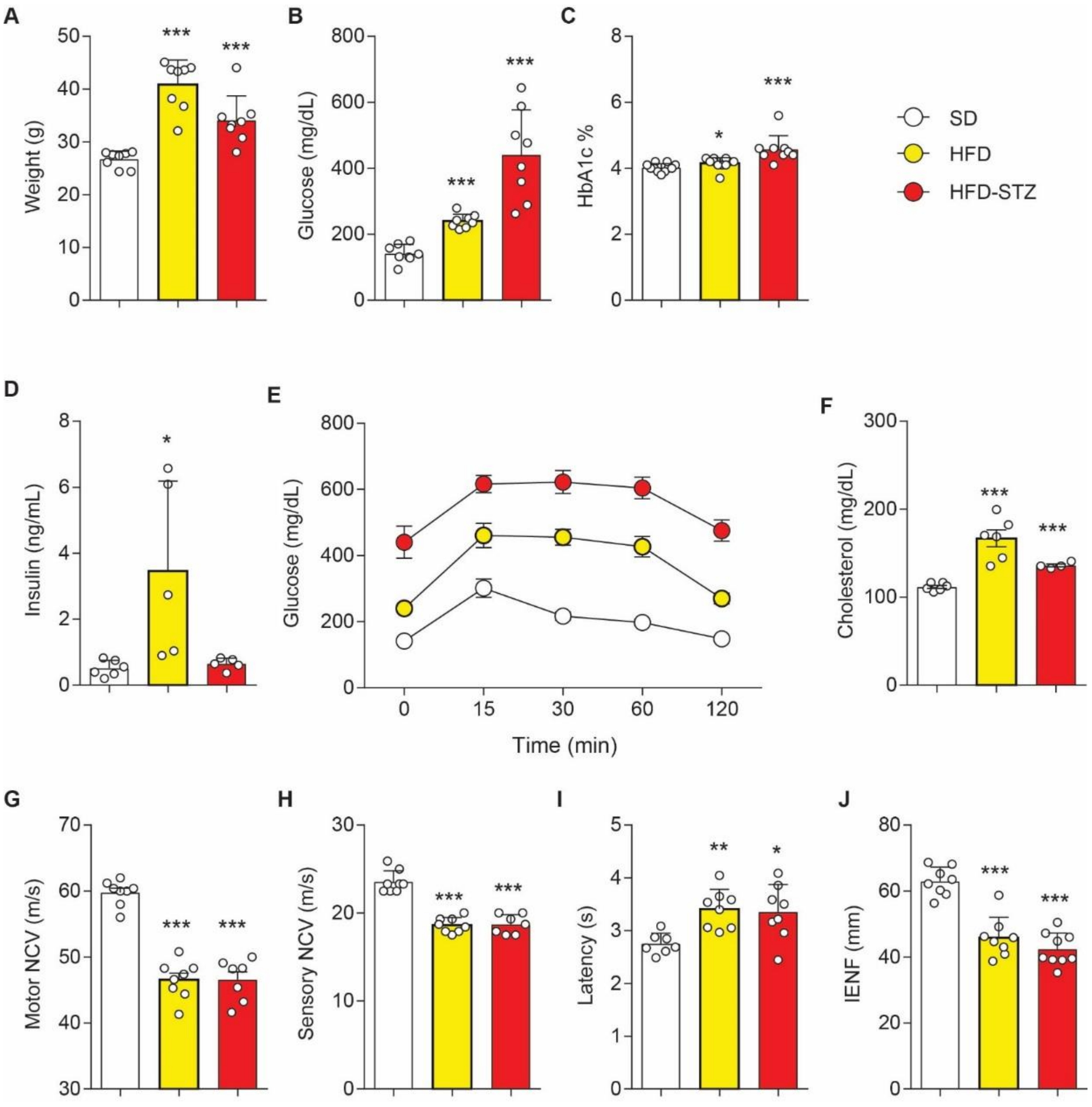

Fig. S1. HFD and HFD-STZ mice develop metabolic deficits and peripheral neuropathy. (A) BW (n = 15-16) (B) FBG (n = 7-8). (C) Percent HbA_{1c} (n = 13-15). (D) Plasma insulin. (E) GTT on 4 h fasted mice at 24 weeks following glucose i.p. injection (2 g/kg; n = 8). (F) Plasma cholesterol (n = 6). (G) Motor NCV (n = 8). (H) Sensory NCV (n = 8). (I) Withdrawal latency to a heat stimulus directed at the hindpaw of the footpad (n = 6-8). (j) IENFD (n = 6-8). All measures above performed at 16 weeks. *$p < 0.05$, **$p < 0.01$, ***$p < 0.001$, one-way ANOVA followed by Tukey's multiple comparisons of diabetic (HFD/HFD-STZ) vs. non-diabetic (SD) mice. All data are the mean ± SEM. BW, body weight; FBG, fasting blood glucose; GTT, glucose tolerance test; NCV, nerve conduction velocity; IENFD, intraepidermal nerve fiber density.

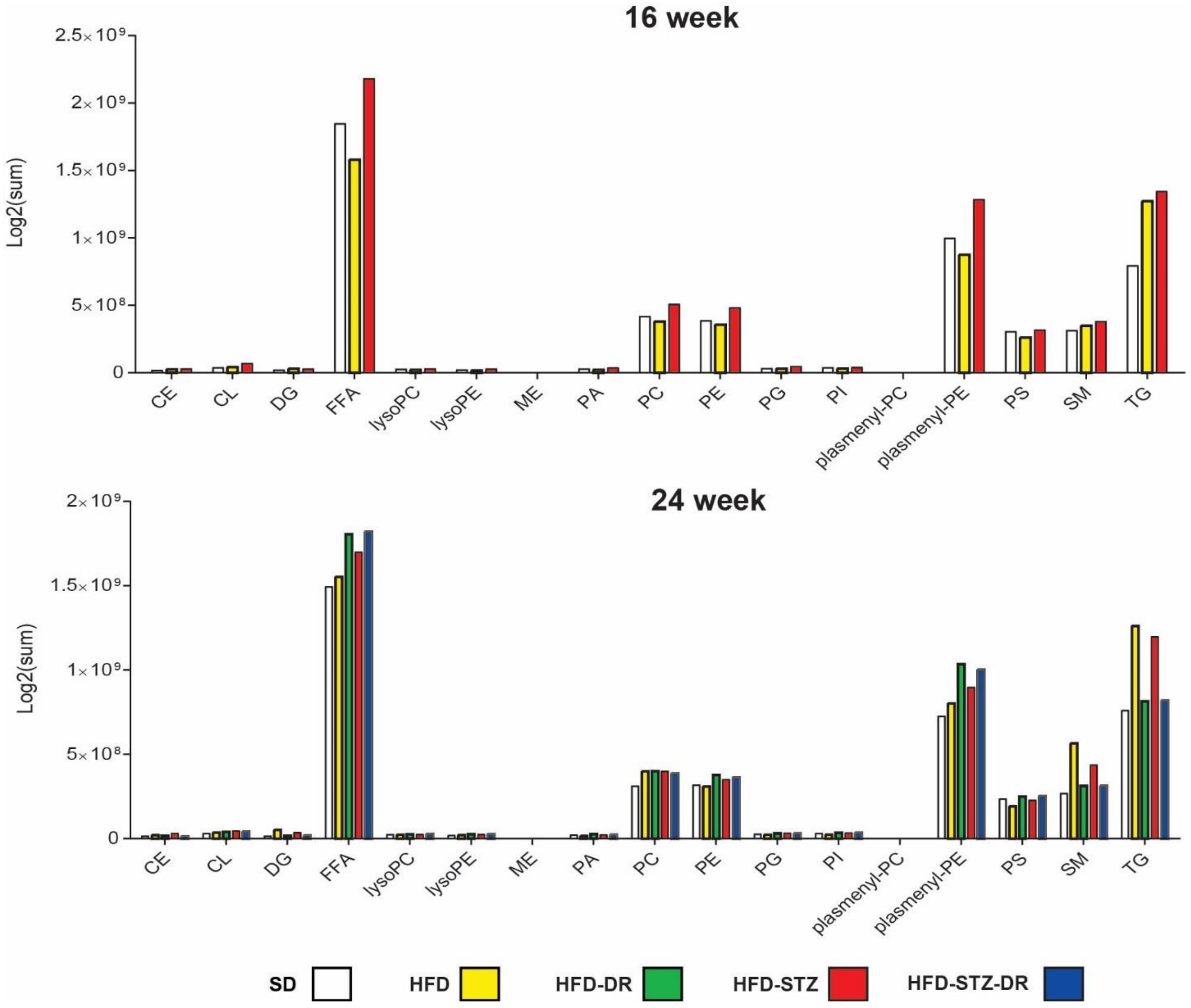

Fig. S2. Altered levels of lipid classes found in SCN tissue. The sum of each lipid species for each class was determined at the 16 (upper panel; before DR) or 24-week (lower panel; after DR) time-point. CE, cholesteryl esters; CL, cardolipins; DG, diglycerides; FFA, free fatty acids; lysoPC, lysophosphatidylcholines; lysoPE, lysophosphatidylethanolamines; ME, methyl esters; PA, phosphatidic acids; PC, phosphatidylcholines; PE, phosphatidylethanolamines; PG, phosphatidylglycerols; PI, phosphatidylinositols; plasmenyl-PC, plasmenyl-phosphatidylcholines; plasmenyl-PE, plasmenyl-phosphatidylethanolamines; PS, phosphatidylserines; SM, sphingomyelins; TG, triglycerides.

Disease Models & Mechanisms • Supplementary information

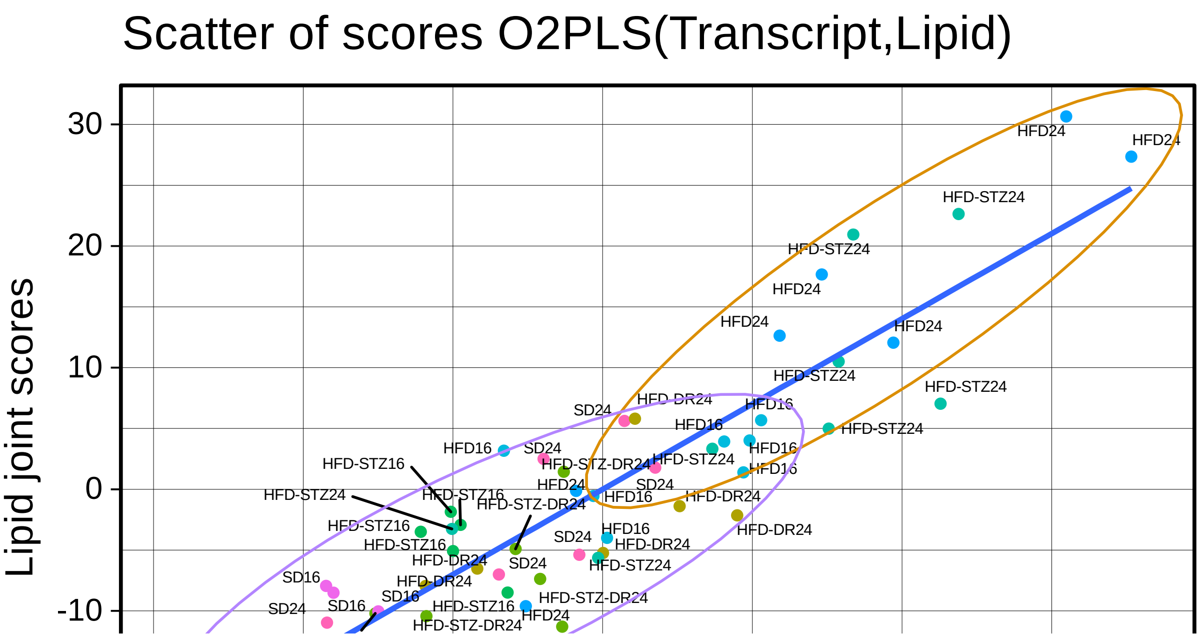

Fig. S3. O2PLS joint score scatter plot. Predicted interaction between the lipid (y-axis) and transcript (x-axis) datasets of joint lipid-scores and transcript-scores. The slope of the fitted line is 0.561, the coefficient of determination R^2 is 0.798. The samples are largely clustered into two groups indicated by two ellipses: one including diabetic/prediabetic groups (HFD16, HFD24, and HFD-STZ24) and non-diabetic/dietary reversal groups (SD16, SD24, HFD-DR24, and HFD-STZ-DR24).

Disease Models & Mechanisms • Supplementary information

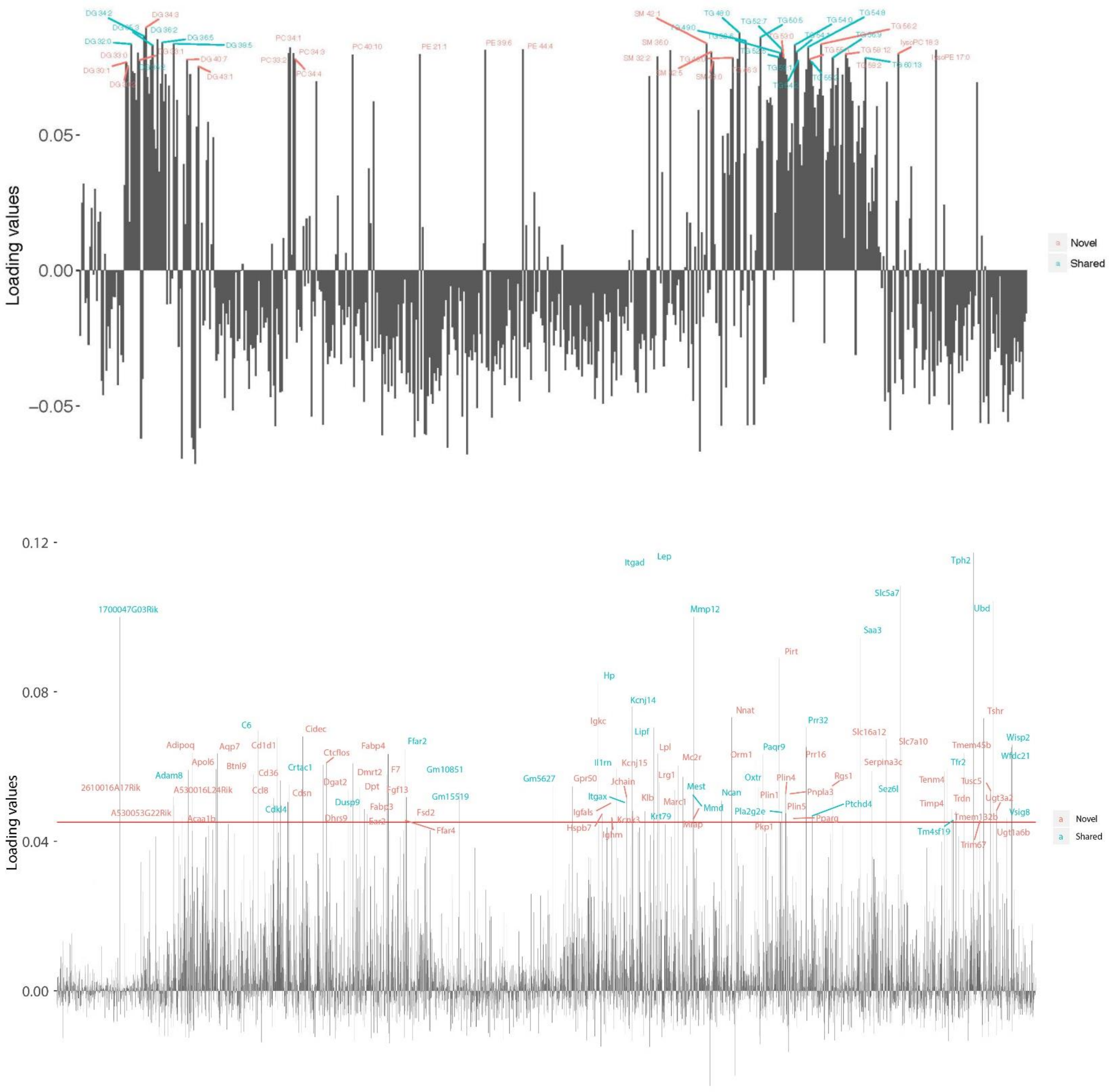

Fig. S4. O2PLS loading plots. O2PLS lipid (top panel) and gene transcript (bottom panel) joint loading plots with the top 50 lipids and 100 genes being highlighted, respectively. The labels in light red represent novel species identified by O2PLS, while the remainder in turquoise were previously identified as DR-reversed DALs/DEGs in our differential analysis. DAL: differentially altered lipid; DEG: differentially expressed gene.

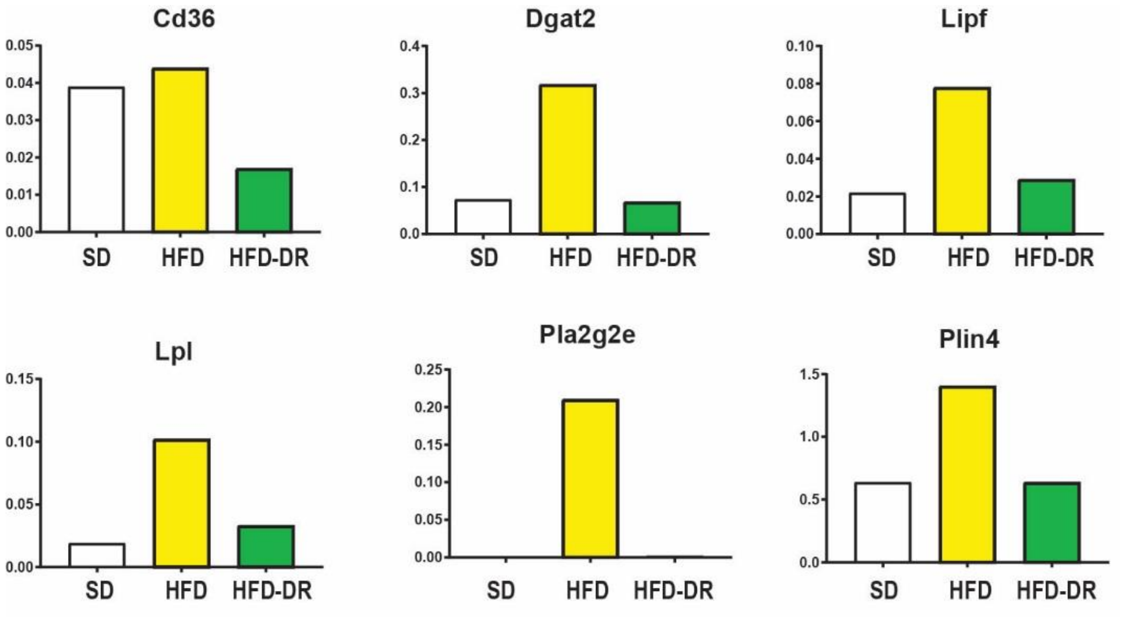

Fig. S5. qPCR validation. Validation of six target genes identified by O2PLS was performed by quantitative real-time qPCR using the pooled RNA samples subjected to RNA-seq (n = 6-10/group). cDNA was generated from 50 ng of pooled RNA, of which 2.5 ng was used for the subsequent qPCR reaction. qPCR was performed in triplicate using sequence specific primers (Table S1). Expression of each gene was calculated from a cDNA titration within each plate (standard curve method) and normalized to the endogenous reference gene, *Ywhaz*.

Disease Models & Mechanisms • Supplementary information

Table S1. Fatty acid composition of standard and high fat diets. The fatty acid profile of the SD, HFD, and HFD-MUFA used to evaluate the role of dietary SFA and MUFA on neuropathy. The total amount of SFA, MUFA, and PUFA in each chow is listed within the table.

	**Standard diet** D12450B (10% kcal fat)	**HFD** D12492 (60% kcal fat)
C10:0, Capric	0	0.1
C12:0, Lauric	0	0.2
C14:0, Myristic	0.3	2.8
C15:0	0	0.2
C16:0, Palmitic	6.4	49.9
C16:1, Palmitoleic	0.3	3.4
C17:0	0.1	0.9
C18:0, Steric	3.1	26.9
C18:1, Oleic	12.3	86.3
C18:2, Linoleic	17.8	72.7
C18:3, Linolenic	2.1	5.1
C20:0, Arachidic	0.1	0.5
C20:1	0.2	1.6
C20:2	0.2	2.0
C20:3	0	0.3
C20:4, Arachidonic	0.1	0.7
C22:0, Behenic	0.1	0.1
C22:5, Docosapentaenoic	0	0.2
Total (g)	43.1	254
Saturated (g)	10.1	81.7
Monounsaturated (g)	12.8	91.2
Polyunsaturated (g)	20.2	81.0
Saturated (%)	23.5	32.2
Monounsaturated (%)	29.7	35.9
Polyunsaturated (%)	46.8	31.9

MUFA, monounsaturated fatty acid; PUFA, polyunsaturated fatty acid; SFA, saturated fatty acid.

Disease Models & Mechanisms • Supplementary information

Table S2. Loading values for top 50 highly intercorrelated lipid species

Species	Loading value	Type	Species	Loading value	Type
DG 34:3	8.97E-02	Novel	PC 34:3	8.01E-02	Novel
TG 48:0	8.78E-02	Shared	lysoPC 18:3	8.01E-02	Novel
DG 34:2	8.65E-02	Shared	TG 58:12	7.99E-02	Novel
TG 50:5	8.61E-02	Shared	PE 21:1	7.99E-02	Novel
DG 36:2	8.53E-02	Shared	PC 40:10	7.97E-02	Novel
TG 49:0	8.48E-02	Shared	TG 53:0	7.89E-02	Novel
DG 36:5	8.42E-02	Shared	SM 32:2	7.89E-02	Novel
SM 42:1	8.39E-02	Novel	TG 52:5	7.87E-02	Shared
DG 38:5	8.37E-02	Shared	TG 46:0	7.86E-02	Novel
TG 56:2	8.36E-02	Novel	TG 56:9	7.85E-02	Shared
DG 32:0	8.36E-02	Shared	TG 60:13	7.85E-02	Shared
TG 54:0	8.33E-02	Shared	TG 58:2	7.83E-02	Novel
DG 35:3	8.31E-02	Shared	PC 34:4	7.81E-02	Novel
TG 54:8	8.25E-02	Shared	TG 46:3	7.81E-02	Novel
PC 34:1	8.23E-02	Novel	TG 53:1	7.80E-02	Shared
TG 52:7	8.15E-02	Shared	TG 55:1	7.80E-02	Novel
PE 44:4	8.15E-02	Novel	DG 40:7	7.79E-02	Novel
lysoPE 17:0	8.14E-02	Novel	DG 35:2	7.77E-02	Shared
PE 39:6	8.14E-02	Novel	DG 33:1	7.76E-02	Novel
SM 36:0	8.12E-02	Novel	TG 54:2	7.74E-02	Shared
SM 42:5	8.07E-02	Novel	SM 43:0	7.72E-02	Novel
TG 54:1	8.06E-02	Shared	TG 55:2	7.71E-02	Shared
DG 33:0	8.04E-02	Novel	DG 30:1	7.69E-02	Novel
TG 52:6	8.03E-02	Shared	DG 30:2	7.55E-02	Novel
PC 33:2	8.03E-02	Novel	DG 43:1	7.53E-02	Novel

DG, diglycerides; lysoPC, lysophosphatidylcholines; lysoPE, lysophosphatidylethanolamines; PC, phosphatidylcholines; PE, phosphatidylethanolamines; SM, sphingomyelins; TG, triglycerides.

Disease Models & Mechanisms • Supplementary information

Table S3. Loading values for top 100 highly inner associated genes

Gene	Loading Value	Type	MGI Gene/Marker ID	Name	Feature Type
Tph2	0.117	Shared	MGI:2384574	transmembrane protein 45b	protein coding gene
Lep	0.114	Shared	MGI:104663	leptin	protein coding gene
Itgad	0.113	Shared	MGI:3578624	integrin, alpha D	protein coding gene
Slc5a7	0.108	Shared	MGI:2147716	solute carrier family 16 (monocarboxylic acid transporters), member 12	protein coding gene
Ubd	0.104	Shared	MGI:1344410	ubiquitin D	protein coding gene
Mmp12	0.100	Shared	MGI:97005	matrix metallopeptidase 12	protein coding gene
1700047G03Rik	0.100	Shared	MGI:1920589	RIKEN cDNA 1700047G03 gene	lincRNA gene
Saa3	0.094	Shared	MGI:1354694	regulator of G-protein signaling 1	protein coding gene
Pirt	0.089	Novel	MGI:2443635	phosphoinositide-interacting regulator of transient receptor potential channels	protein coding gene
Hp	0.083	Shared	MGI:96211	haptoglobin	protein coding gene
Kcnj14	0.076	Shared	MGI:2384820	potassium inwardly-rectifying channel, subfamily J, member 14	protein coding gene
Igkc	0.075	Novel	MGI:96495	immunoglobulin kappa constant	gene segment
Nnat	0.073	Novel	MGI:104716	neuronatin	protein coding gene
Tshr	0.073	Novel	MGI:98849	thyroid stimulating hormone receptor	protein coding gene
Prr32	0.071	Shared	MGI:1918623	proline rich 16	protein coding gene
Lipf	0.070	Shared	MGI:1914967	lipase, gastric	protein coding gene
C6	0.070	Shared	MGI:88233	complement component 6	protein coding gene
Cidec	0.068	Novel	MGI:95585	cell death-inducing DFFA-like effector c	protein coding gene
Cd1d1	0.068	Novel	MGI:107674	CD1d1 antigen	protein coding gene
Slc16a12	0.067	Novel	MGI:1935121	seizure related 6 homolog like	protein coding gene
Wisp2	0.066	Shared	MGI:1328326	WNT1 inducible signaling pathway protein 2	protein coding gene
Prr16	0.065	Novel	MGI:1890505	perilipin 1	protein coding gene
Wfdc21	0.065	Shared	MGI:1913357	WAP four-disulfide core domain 21	protein coding gene
Slc7a10	0.065	Novel	MGI:1927126	solute carrier family 5 (choline transporter), member 7	protein coding gene
Ffar2	0.065	Shared	MGI:2441731	free fatty acid receptor 2	protein coding gene
Adipoq	0.064	Novel	MGI:106675	adiponectin, C1Q and collagen domain containing	protein coding gene
Tmem45b	0.064	Novel	MGI:1354956	transferrin receptor 2	protein coding gene
Aqp7	0.063	Novel	MGI:1314647	aquaporin 7	protein coding gene
Lpl	0.063	Novel	MGI:96820	lipoprotein lipase	protein coding gene
Fabp4	0.063	Novel	MGI:88038	fatty acid binding protein 4, adipocyte	protein coding gene
Paqr9	0.063	Shared	MGI:1922802	progestin and adipoQ receptor family member IX	protein coding gene
Orm1	0.061	Novel	MGI:97443	orosomucoid 1	protein coding gene
Ctcflos	0.061	Novel	MGI:1921411	CCCTC-binding factor (zinc finger protein)-like, opposite strand	antisense lncRNA gene
Dmrt2	0.061	Novel	MGI:1330307	doublesex and mab-3 related transcription factor 2	protein coding gene
Crtac1	0.060	Shared	MGI:1920082	cartilage acidic protein 1	protein coding gene
Mc2r	0.060	Novel	MGI:96928	melanocortin 2 receptor	protein coding gene
Il1rn	0.059	Shared	MGI:96547	interleukin 1 receptor antagonist	protein coding gene
Tfr2	0.059	Shared	MGI:2447063	teneurin transmembrane protein 4	protein coding gene
Apol6	0.059	Novel	MGI:1919189	apolipoprotein L 6	protein coding gene

Adam8	0.059	Shared	MGI:107825	a disintegrin and metallopeptidase domain 8	protein coding gene
Serpina3c	0.059	Novel	MGI:98223	serum amyloid A 3	protein coding gene
Tenm4	0.059	Novel	MGI:1858261	solute carrier family 7 (cationic amino acid transporter, y+ system), member 10	protein coding gene
Kcnj15	0.059	Novel	MGI:1310000	potassium inwardly-rectifying channel, subfamily J, member 15	protein coding gene
Btnl9	0.058	Novel	MGI:2442439	butyrophilin-like 9	protein coding gene
F7	0.058	Novel	MGI:109325	coagulation factor VII	protein coding gene
Gm10851	0.057	Shared	MGI:3641824	predicted gene 10851	antisense lncRNA gene
Mest	0.057	Shared	MGI:96968	mesoderm specific transcript	protein coding gene
Cd36	0.056	Novel	MGI:107899	CD36 molecule	protein coding gene
Sez6l	0.056	Shared	MGI:102848	serine (or cysteine) peptidase inhibitor, clade A, member 3C	protein coding gene
Lrg1	0.056	Novel	MGI:1924155	leucine-rich alpha-2-glycoprotein 1	protein coding gene
Cdsn	0.055	Novel	MGI:3505689	corneodesmosin	protein coding gene
Rgs1	0.055	Novel	MGI:1916050	proline rich 32	protein coding gene
Gm5627	0.055	Shared	MGI:3645308	predicted gene 5627	protein coding gene
Gpr50	0.055	Novel	MGI:1333877	G-protein-coupled receptor 50	protein coding gene
Dpt	0.054	Novel	MGI:1928392	dermatopontin	protein coding gene
Oxtr	0.054	Shared	MGI:109147	oxytocin receptor	protein coding gene
Dgat2	0.054	Novel	MGI:1915050	diacylglycerol O-acyltransferase 2	protein coding gene
Marc1	0.054	Novel	MGI:1913362	mitochondrial amidoxime reducing component 1	protein coding gene
Tusc5	0.054	Novel	MGI:3029307	tumor suppressor candidate 5	protein coding gene
Trdn	0.054	Novel	MGI:2651811	tryptophan hydroxylase 2	protein coding gene
Gm15519	0.053	Shared	MGI:3782965	predicted gene 15519	pseudogene
Plin1	0.053	Novel	MGI:1890505	perilipin 1	protein coding gene
Plin4	0.053	Novel	MGI:1929709	perilipin 4	protein coding gene
Pnpla3	0.053	Novel	MGI:2151796	patatin-like phospholipase domain containing 3	protein coding gene
2610016A17Rik	0.053	Novel	MGI:1919653	RIKEN cDNA 2610016A17 gene	lincRNA
Mmd	0.052	Shared	MGI:1914718	monocyte to macrophage differentiation-associated	protein coding gene
Jchain	0.052	Novel	MGI:96493	immunoglobulin joining chain	protein coding gene
A530016L24Rik	0.052	Novel	MGI:2443020	RIKEN cDNA A530016L24 gene	protein coding gene
Fgf13	0.052	Novel	MGI:109178	fibroblast growth factor 13	protein coding gene
Ccl8	0.052	Novel	MGI:101878	chemokine (C-C motif) ligand 8	protein coding gene
Ncan	0.051	Shared	MGI:104694	neurocan	protein coding gene
Cdkl4	0.051	Shared	MGI:3587025	cyclin-dependent kinase-like 4	protein coding gene
Itgax	0.050	Shared	MGI:96609	integrin alpha X	protein coding gene
Igfals	0.050	Novel	MGI:107973	insulin-like growth factor binding protein, acid labile subunit	protein coding gene
Klb	0.050	Novel	MGI:1932466	klotho beta	protein coding gene
A530053G22Rik	0.050	Novel	MGI:2443347	RIKEN cDNA A530053G22 gene	lincRNA
Mrap	0.049	Novel	MGI:1924287	melanocortin 2 receptor accessory protein	nonsense-mediated decay
Dusp9	0.049	Shared	MGI:2387107	dual specificity phosphatase 9	protein coding gene
Timp4	0.048	Novel	MGI:109125	tissue inhibitor of metalloproteinase 4	protein coding gene
Ugt3a2	0.048	Novel	MGI:2145969	UDP glycosyltransferases 3 family, polypeptide A2	protein coding gene

Dhrs9	0.048	Novel	MGI:2442798	dehydrogenase/reductase (SDR family) member 9	protein coding gene
Kcnk3	0.048	Novel	MGI:1100509	potassium channel, subfamily K, member 3	protein coding gene
Krt79	0.048	Shared	MGI:2385030	keratin 79	protein coding gene
Tmem132b	0.048	Novel	MGI:3609245	transmembrane protein 132B	protein coding gene
Pla2g2e	0.048	Shared	MGI:1349660	phospholipase A2, group IIE	protein coding gene
Plin5	0.048	Novel	MGI:1914218	perilipin 5	protein coding gene
Fabp3	0.047	Novel	MGI:95476	fatty acid binding protein 3, muscle and heart	protein coding gene
Ear2	0.047	Novel	MGI:108020	eosinophil-associated, ribonuclease A family, member 2	protein coding gene
Hspb7	0.047	Novel	MGI:1352494	heat shock protein family, member 7 (cardiovascular)	protein coding gene
Ptchd4	0.047	Shared	MGI:1920485	patched domain containing 4	protein coding gene
Fsd2	0.046	Novel	MGI:2444310	fibronectin type III and SPRY domain containing 2	protein coding gene
Acaa1b	0.046	Novel	MGI:3605455	acetyl-Coenzyme A acyltransferase 1B	protein coding gene
Ighm	0.046	Novel	MGI:96448	immunoglobulin heavy constant mu	IG C gene
Vsig8	0.046	Shared	MGI:3642995	V-set and immunoglobulin domain containing 8	protein coding gene
Pparg	0.046	Novel	MGI:97747	peroxisome proliferator activated receptor gamma	protein coding gene
Tm4sf19	0.046	Shared	MGI:3645933	transmembrane 4 L six family member 19	protein coding gene
Ffar4	0.046	Novel	MGI:2147577	free fatty acid receptor 4	protein coding gene
Trim67	0.045	Novel	MGI:3045323	tripartite motif-containing 67	protein coding gene
Pkp1	0.045	Novel	MGI:1328359	plakophilin 1	protein coding gene
Ugt1a6b	0.045	Novel	MGI:3580629	UDP glucuronosyltransferase 1 family, polypeptide A6B	protein coding gene

Table S4. Clinical characteristics of subjects separated by HbA_{1C} status

	Gender	Age (yrs)	BMI (kg/m^2)	Diabetes duration (yrs)	HbA_{1c} % (mmol/mol)	Triglycerides (mmol/L)	Cholesterol (mmol/L)	Basal MFD (fibers/mm^2)
Low HbA_{1C}	Male	53	23.1	14.089	6.3	0.69	3.6	5121.63
	Male	63	25.2	3.4661	6.7	2.9	6.5	4231.2
	Male	61	25.4	1.2704	6.2	0.66	3.9	1978.83
	Male	70	30.2	10.642	6.3	2.19	3.9	5460.59
	Male	42	40.3	2.7214	6.4	2.07	5.8	5662.14
	Male	65	25.7	2.0726	6.4	1.82	6.4	5159.32
	Female	59	27.8	1.2266	5.9	3.19	6.2	5934.81
	Male	69	22.3	18.0972	6	1.06	4	1014.03
	Female	52	27.7	6.0589	6	1.3	7	2312.36
High HbA_{1c}	Female	69	26.4	8.167	10.2	1.62	5.1	4893.19
	Male	62	30	7.7016	10.5	1.85	4.4	1917.38
	Male	42	32	18.7488	11.4	1.71	5.1	1994.25
	Male	43	33	2.768	9.7	2.08	5.1	6234.79
	Female	45	29.4	9.7358	12.6	4.41	6.9	2835.25
	Female	68	30	8.6516	12.7	2.92	5.4	1833.65
	Female	48	37.8	4.0575	12.3	1.95	5.1	48.42

**Summary of subject characteristics separated by HbA_{1C} status**			
	Controlled	Uncontrolled	p-value (t-test)
Age (yrs)	59.33±9.01	53.86±12.02	0.3366
Gender	7 Males, 2 Females	3 Males, 4 Females	
BMI (kg/m^2)	27.52±5.37	31.23±3.57	0.1209
Diabetes duration (yrs)	6.63±6.2	8.55±5.16	0.5104
HbA_{1c} % (mmol/mol)	6.24±0.25	11.34±1.23	**2.43E-05****
Cholesterol (mmol/L)	5.26±1.37	5.3±0.77	0.2404
Triglycerides (mmol/L)	1.76±0.91	2.36±1	0.9359
Basal MFD (fibers/mm^2)	4097.2±1839.1	2822.4±2086.1	0.2153

MFD, myelin fiber density.

Disease Models & Mechanisms • Supplementary information

Table S5. Clinical characteristics of subjects separated by lipid status

	Gender	Age (yrs)	BMI (kg/m^2)	Diabetes duration (yrs)	HbA1c % (mmol/mol)	Triglycerides (mmol/L)	Cholesterol (mmol/L)	Basal MFD ($fibers/mm^2$)
Low Lipids	Male	65	29.2	5.0623	6	1.1	4.2	6549.34
	Female	70	23.3	8.6297	7.8	1.06	5.2	4849.15
	Male	63	32.7	2.4285	7.8	1.33	4.6	5296.07
	Male	35	23.3	7.5236	8	0.61	3.4	5066.99
	Male	63	28.9	9.5606	8	0.89	4.3	E
	Male	56	33.3	11.5893	6.7	0.86	4	4724.3
	Male	55	25	12.7529	10.1	0.72	4.3	2330.36
	Male	62	30.4	9.5852	6.9	1.51	4.2	3395.54
	Male	42	21.7	7.4442	7.4	0.52	4.3	4080.57
	Female	52	30	21.9767	6.3	0.97	4.2	4968.85
High Lipids	Female	68	24.1	8.0739	7.1	3.39	7.9	4091.17
	Female	57	25	10.5161	9.1	3.74	7	3402.67
	Female	69	30.3	10.6064	6	6.29	6.9	2524.14
	Male	48	33.1	10.5435	12.2	3.53	7.7	3557.78
	Male	49	33.8	5.7906	10.5	8.58	5.4	3240.44
	Male	57	24.2	4.0055	6.4	8.94	8.4	3582.93
	Female	69	22.9	8.3422	6.5	4.2	7.3	3130.69
	Female	54	27.9	6.475	7.6	14.75	9.2	1583.32
	Female	64	28.2	25.9329	6.8	3.76	6.9	1843.26
	Male	63	33.4	6.7159	7.5	4.02	7.3	2104.67

**Summary of Subjects Characteristics separated by Lipid Status**			
	Normal	High	p-value (t-test)
Age (yrs)	56.3±10.87	59.8±7.96	0.4231
Gender	4 Males, 6 Females	8 Males, 2 Females	
BMI (kg/m^2)	27.78±4.14	28.29±4.19	0.7875
Diabetes duration (yrs)	9.66±5.26	9.7±6.12	0.9862
HbA_{1c} % (mmol/mol)	7.5±1.16	7.97±2.02	0.5329
Cholesterol (mmol/L)	4.27±0.45	7.4±1.01	**9.46E-07****
Triglycerides (mmol/L)	0.96±0.31	6.12±3.68	**1.62E-03****
Basal MFD ($fibers/mm^2$)	4584.6±1203.4	2906.1±840.9	**2.4E-03****

MFD, myelin fiber density.

Disease Models & Mechanisms • Supplementary information

Table S6. Lipid species identified in SCN tissue

Table displaying the raw data for each lipid species using tandem mass spectrometry (LC-MS/MS) untargeted lipidomics of SCN at the 16 or 24-week time-point.

CE, cholesteryl esters; CL, cardolipins; DG, diglycerides; FFA, free fatty acids; lysoPC, lysophosphatidylcholines; lysoPE, lysophosphatidylethanolamines; ME, methyl esters; PA, phosphatidic acids; PC, phosphatidylcholines; PE, phosphatidylethanolamines; PG, phosphatidylglycerols; PI, phosphatidylinositols; plasmenyl-PC, plasmenyl-phosphatidylcholines; plasmenyl-PE, plasmenyl-phosphatidylethanolamines; PS, phosphatidylserines; SM, sphingomyelins; TG, triglycerides.

Click here to Download Table S6

Table S7. qPCR mouse primer sequences

Gene Symbol	Gene ID#	Primer Sequence	
Ywhaz	22631	Forward	5′-AAGACAGCACGACGCTAATAATGC
		Reverse	5′-TTGGAAGGCCGGTTAATTTTC
Cd36	12491	Forward	5′-TTCTTCACAGCTGCCTTCTGA
		Reverse	5′-TTTCTACGTGGCCCGGTTCTAA
Dgat2	67800	Forward	5′-ACACCTTCTGCACAGACTGC
		Reverse	5′-TGCGATCTCCTGCCACCTTT
Lipf	67717	Forward	5′-GATGCTGGCTATGATGTGTGGC
		Reverse	5′-GTCTATGGTGGCTGGAAGGTCA
Lpl	16956	Forward	5′-TGGCGTAGCAGGAAGTCTGA
		Reverse	5′-TGCCTCCATTGGGATAAATGTC
Pla2g2e	26970	Forward	5′-ATGAAACCTCCCATTGCCCT
		Reverse	5′-GGGCATGACAACACCAATCC
Plin4	57435	Forward	5′-CTGAACAGACAGCTGGAGACA
		Reverse	5′-CTTTGGCCACGCCCACT

Disease Models & Mechanisms • Supplementary information
